# Mechanisms of alkaliptosis

**DOI:** 10.3389/fcell.2023.1213995

**Published:** 2023-08-04

**Authors:** Fangquan Chen, Rui Kang, Jiao Liu, Daolin Tang

**Affiliations:** ^1^ The Third Affiliated Hospital of Guangzhou Medical University, Guangzhou, Guangdong, China; ^2^ Department of Surgery, UT Southwestern Medical Center, Dallas, TX, United States

**Keywords:** alkaliptosis, cancer, cell death, immunity, pH

## Abstract

Malignant tumors represent a major threat to global health and the search for effective treatments is imperative. While various treatments exist, including surgery, radiotherapy, chemotherapy, immunotherapy and combination therapies, there remains a need to develop therapies that target regulated cell death pathways to eliminate cancer cells while preserving normal cells. Alkaliptosis, a pH-dependent cell death process triggered by the small molecular compound JTC801, has been identified as a novel approach for malignant tumor treatment, particularly in pancreatic cancer. Two major signaling pathways, the NF-κB-CA9 pathway and the ATP6V0D1-STAT3 pathway, contribute to the induction of alkaliptosis. This review summarizes recent developments in our understanding of alkaliptosis signals, mechanisms, and modulation, and explores its context-dependent effects on drug resistance, inflammation, and immunity. By providing a deeper understanding of the heterogeneity and plasticity of cell death mechanisms, this information holds promise for informing the design of more effective anti-tumor therapies.

## Introduction

Cell death is one group of mechanisms of the end of cell life and can occur quickly or gradually. Characteristic morphological changes, such as alterations in the nucleus, mitochondria and cell volume, often accompany cell death. The diversity of causes leads to differing degrees of morphological changes during the process of cell death (Ziegler and Groscurth, 2004). Functionally, cell death can be divided into normal and abnormal death, with normal death being a part of physiological development and abnormal death resulting from environmental stress or pathological conditions (Green and Llambi, 2015). Mechanically, cell death can be categorized into accidental cell death (ACD) and regulated cell death (RCD) (Galluzzi et al., 2018). ACD is a biologically uncontrolled process, while RCD is a precisely regulated signaling pathway with specific effectors. The subtypes of regulated cell death (RCD) include apoptosis-dependent and non-apoptosis-dependent, and each displays unique morphological, biochemical, and genetic features (Tang et al., 2019; Ajoolabady et al., 2021).

Cancer is a significant contributor to global economic loss due to its impact on premature death, disability, and cost of treatment. Ongoing efforts are being made to find effective treatments for cancer. Currently, available cancer treatments include surgery, radiotherapy, chemotherapy, immunotherapy, and combination therapies, all of which aim to remove, shrink, or suppress the growth of cancer cells. These treatments can be applied as primary treatment to completely remove or kill all cancer cells from the body, as adjuvant therapy to eliminate residual cancer cells following primary treatment, or as palliative care to manage the adverse effects of treatment and symptoms associated with the cancer (Schirrmacher, 2019). Due to the primary or secondary resistance of tumor cells to apoptosis, stimulating non-apoptotic cell death (e.g., ferroptosis and necroptosis) is an emerging trend in cancer research (Najafov et al., 2017; Chen et al., 2021a; Chen et al., 2021b; Liu et al., 2021; Tang et al., 2023a; Zhang et al., 2023).

The dysregulation of pH is a frequent hallmark of cancer cells, characterized by elevated intracellular pH and reduced extracellular pH compared to normal cells (Neri and Supuran, 2011; Cassim and Pouyssegur, 2019; Parks et al., 2020). The disruption of pH homeostasis in cancer cells is expected to inhibit tumor growth and progression (Ward et al., 2020). Our group, in 2018, identified a novel form of pH-dependent RCD, known as alkaliptosis, displaying potent anti-tumor efficacy against various cancer types, particularly pancreatic ductal adenocarcinoma (PDAC) (Song et al., 2018). Compared to other forms of RCD, the induction of alkaliptosis does not involve the commonly recognized cell death effectors, such as caspases and mixed lineage kinase domain like pseudokinase (MLKL) (Song et al., 2018). Furthermore, the inducer of alkaliptosis exhibits minimal toxicity towards normal cells or tissues (Song et al., 2018). Alkaliptosis induction has been shown to suppress tumors in a range of mouse models, including xenografts, orthotopic tumors, metastatic tumors, and transgenic models (Song et al., 2018).

In this review, we provide an overview of the latest insights into the mechanism of alkaliptosis and focus on its impact on tumor properties, such as drug resistance and tumor immunity.

## The discovery of alkaliptosis

Pancreatic cancer is a gastrointestinal malignancy characterized by a high mortality rate, primarily attributed to the frequent challenges in diagnosing it at a late stage and its resistance to therapy (Kang et al., 2017; Li et al., 2021; Siegel et al., 2022). The exocrine form of PDAC, which accounts for 95% of cases, is regulated by a complex interplay of internal and external factors and characterized by genetic mutations, such as *KRAS* mutations, and a challenging immune microenvironment (Karamitopoulou, 2019; Buscail et al., 2020). Despite recent advancements in targeting stromal responses and immune evasion, and the development of drugs to inhibit KRAS-G12C (e.g., sotorasib/AMG 510) and KRAS-G12D (e.g., MRTX1133) protein, many patients remain unresponsive to these therapies (Hong et al., 2020; Skoulidis et al., 2021; Liu et al., 2022a; Hallin et al., 2022; Wang et al., 2022; Tang and Kang, 2023). Further understanding of the molecular pathology of PDAC and the development of new drugs are crucial in improving the prognosis of PDAC patients (Neoptolemos et al., 2018; Chen et al., 2021c).

G protein-coupled receptors (GPCRs), a class of membrane proteins with 7 transmembrane helices and over 800 family members, play crucial roles in regulating biological processes and are, therefore, a common drug target (Hauser et al., 2017). To find an effective chemotherapy agent for treating PDAC with *KRAS-G12D* mutations, a library of GPCR-targeted drug molecules was tested in human PDAC cell lines, mouse pancreatic cancer-associated stellate cell lines, and 60 different human tumor cell lines (Song et al., 2018). The small molecule compound JTC801 is identified as having strong cancer cell-killing effects. Further *in vivo* experiments in mice showed that JTC801 selectively targets PDAC cells without harming normal cells (Song et al., 2018). Subsequent chemical structure and functional studies confirmed that JTC801 induces a new form of cell death known as alkaliptosis (Song et al., 2018).

JTC801 is a selective antagonist for the nociceptin receptor, also referred to as opioid-related nociceptin receptor 1 (OPRL1) (Muratani et al., 2002). However, the induction of alkaliptosis by JTC801 is not contingent on the OPRL1 receptor. JTC801-induced cell death does not rely on apoptosis, necroptosis, or ferroptosis, as no significant induction of their molecular markers are observed (e.g., caspase 3 or poly [ADP ribose] polymerase [PARP] cleavage for apoptosis, MLKL and receptor-interacting serine-threonine kinase 3 [RIPK3] for necroptosis, glutathione peroxidase 4 (GPX4) degradation for ferroptosis). Additionally, blocking these processes through RNAi or inhibitors does not prevent JTC801-induced cell death (Song et al., 2018).

Autophagy is a lysosomal degradation mechanism that either promotes or hinders cell death in response to stress signals (Doherty and Baehrecke, 2018). Despite induction by JTC801, blocking autophagy through the deletion of autophagy-related genes (*Atg3* ^*−/−*^, *Atg5* ^*−/−*^ *, Atg7* ^*−/−*^, or *Sqstm1* ^*−/−*^) does not prevent alkaliptosis (Song et al., 2018). In contrast, alkaliptosis induced by JTC801 in PDAC cells is reversed in acidic conditions (pH 6.2) or with N-acetyl cysteine or N-acetyl alanine (Table 1), highlighting alkalinization as a mediator of alkaliptosis.

**TABLE 1:** Potential modulators and inhibitors of alkaliptosis.

Targets	Functions	Inhibitors (targets)
IKBKB	Inhibiting CA9 expression	IMD0354 (IKBKB), CAY10657 (IKBKB)
CA9	Inhibiting cytosolic alkalization	SC514 (IKBKB), Bafilomycin A1 (V-ATPase)
ATP6V0D1	Promoting cytosolic proton pump into lysosomes or export to extracellular	N-acetyl cysteine (pH)
ACSS2	Promoting NF-κB acetylation	N-acetyl alanine acid (pH)
SLC9A1	Promoting extracellular proton transport into cells	
SLC9A7	Promoting extracellular proton transport into cells or pump into the Golgi apparatus	
TMEM175	Promoting lysosomal proton efflux	
SLC4A4	Promoting bicarbonate transfer into cells	
SLC7A5	Promoting intracellular uptake of methionine	

Collectively, these findings suggest that the mechanism of alkaliptosis involves unique molecules (to be discussed later).

## The mechanism of alkaliptosis

**FIGURE 1:**
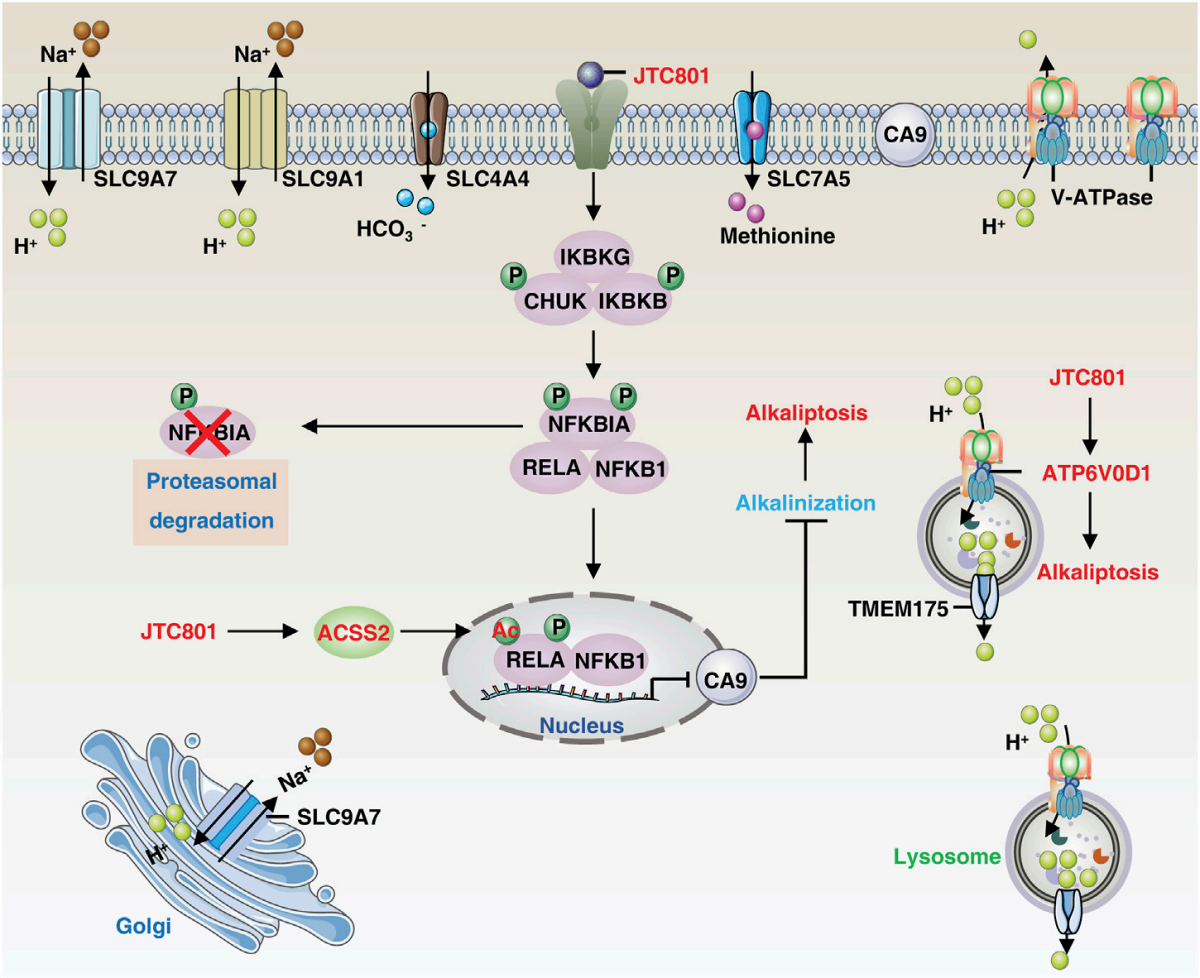
The core molecular mechanisms of alkaliptosis. The molecular mechanisms underlying alkaliptosis involve pH-induced alkalization, mainly through the activation of JTC801, which promotes the transcriptional repression of CA9 by NF-κB. JTC801 also induces acetylation of NF-κB by ACSS2, leading to alkaliptosis. The action of JTC801 results in the acidification of lysosomes or the extracellular environment, resulting in cytosolic alkalinization. Conversely, TMEM175 mediates lysosomal proton transport to the cytosol. The suppression of pH regulators, such as SLC9A7 or SLC9A1, also results in alkalization-related cell death. Targeting SLC4A4 or SLC7A5 can also induce pH-dependent cell death. Abbreviation. IKBKB, inhibitor of nuclear factor-κB kinase subunit-β; ATP6V0D1, ATPase H^+^ transporting V0 subunit D1; SLC9A1, solute carrier family 9 member A1; SLC9A7, solute carrier family 9 member A7; TMEM175, transmembrane protein 175; RELA, RELA proto-oncogene, NF-KB subunit; IKBKG, inhibitor of nuclear factor kappa B kinase regulatory subunit gamma; CHUK, component of inhibitor of nuclear factor kappa B kinase complex; NFKBIA, NFKB inhibitor alpha; NFKB1, nuclear factor kappa B subunit 1; CA9, carbonic anhydrase 9; ACSS2, acyl-CoA synthetase short chain family member 2; SLC7A5, solute carrier family 7 member 5; SLC4A4, solute carrier family 4 member 4.

Alkaliptosis is defined by the lethal rise in intracellular pH, regulated by the interplay of ion channels and transporters in intracellular and extracellular pathways. This section summarizes the existing and potential mechanisms underlying alkaliptosis regulation (Figure 1).

### The NF-κB-CA9 pathway

Nuclear factor-κB (NF-κB) is a family of inducible transcription factors that promote or repress the expression of numerous genes involved in a variety of processes, including immunity, cell survival, and cell death (Taniguchi and Karin, 2018). Mechanically, JTC801 induces alkaliptosis by activating NF-κB-dependent carbonic anhydrase 9 (CA9) downregulation. CA9 catalyzes the reversible hydration of carbon dioxide and is overexpressed in many types of solid cancer, including clear cell renal cell carcinoma and PDAC, where it promotes tumor growth by maintaining acidosis (Swietach et al., 2007). JTC801 activates NF-κB in the same pattern as lipopolysaccharide, a component of Gram-negative bacteria. JTC801-induced NF-κB activation events include activation of the IKK protein complex [including inhibitor of nuclear factor kappa B kinase complex (CHUK, also known as IKKα), nuclear factor kappa B kinase subunit beta (IKBKB, also known as IKKβ), and inhibitor of nuclear factor kappa B kinase regulatory subunit gamma (IKBKG, also known as IKKγ)] through the phosphorylation and degradation of NFKB inhibitor alpha (NFKBIA, also known as IκBα), resulting in the nuclear translocation of nuclear factor kappa B subunit 1 (NFKB1, also known as p50) and RELA proto-oncogene (RELA, also known as p65). Thus, the inhibition of IKKβ by genetic or pharmacological means effectively prevents alkaliptosis (Song et al., 2018; Liu et al., 2020).

In addition to phosphorylation, protein acetylation modification plays a role in the regulation of alkaliptosis. The process is mediated by acyl-CoA synthetase short-chain family member 2 (ACSS2), which produces acetyl-CoA and results in NF-κB acetylation and subsequent activation of the NF-κB pathway, promoting alkaliptosis in PDAC cells (Que et al., 2023). These findings also establish a link between fatty acid synthesis and increased sensitivity to alkaliptosis. As NF-κB is known to play a pro-survival role in drug resistance, inducing NF-κB-dependent cell death, specifically alkaliptosis, offers new avenues for the development of anticancer strategies.

### The ATP6V0D1-STAT3 pathway

The presence of OPRL1, a known target of JTC801, is not essential for alkaliptosis (Chen et al., 2022c). Mass spectrometry-based drug target identification in JTC801-treated human PANC1 cells shows that ATPase H^+^ transporting V0 subunit D1 (ATP6V0D1) acts as a direct target of JTC801 to promote alkaliptosis independent of the NF-κB-CA9 pathway. ATP6V0D1, a member of the vacuolar ATPase (V-ATPase) family, regulates the acidification of intracellular organelles. It does so by forming a complex with a signal transducer and activator of transcription 3 (STAT3) in lysosomes, leading to lysosomal acidification and cytosolic alkalinization, thus inducing alkaliptosis (Chen et al., 2022c). Inhibiting ATP6V0D1 expression via shRNA or mutating the JTC801-binding site of ATP6V0D1 (ARG218) reverses JTC801-induced alkaliptosis (Chen et al., 2022c). However, inhibiting ATP6V0D1 fails to prevent JTC801-induced lysosomal acidification, indicating the potential involvement of a compensatory mechanism (Mindell, 2012; Hu et al., 2022a). Indeed, V-ATPases, in conjunction with various ion transporters or exchangers, constitute a regulatory network responsible for maintaining the internal steady-state pH of the lysosome. Transmembrane protein 175 (TMEM175) has been shown to act as a direct proton exporter to maintain lysosomal pH balance when lysosomal pH drops below 4.6 (Hu et al., 2022b).

Cancer cells, due to their increased glycolytic flux, produce excess acid that can be expelled outside the cell or pumped into organelles (Webb et al., 2011). For example, inhibiting solute carrier family 9 member A7 (SLC9A7, also known as NHE7) induces Golgi alkalization, causing cytosolic acidification and abolishing pancreatic ductal adenocarcinoma tumor growth (Galenkamp et al., 2020). Similarly, osmotic stress can activate plasma membrane solute carrier family 9 member A1 (SLC9A1, also known as NHE1), resulting in increased cytosolic pH and activation of MLKL-mediated necroptosis (Zhang W. et al., 2022). STAT3 is required for SLC9A family expression in some contexts (Su et al., 2009), suggesting a role for nuclear STAT3 in promoting alkaliptosis by mediating the plasma membrane rupture. Regardless, STAT3 is a validated anticancer target that promotes tumorigenesis (Huynh et al., 2019). STAT3-overactive cancers can activate drug resistance genes like antiapoptotic BCL2, but inducing STAT3-dependent alkaliptosis may be a rational strategy to overcome this resistance (Chen et al., 2022c). The maintenance of a reversed pH gradient via solute carrier family 4 member 4 (SLC4A4) expression is essential for glycolysis-dependent lactate production and immune evasion in PDAC (Cappellesso et al., 2022). Conversely, acid exposure inhibits the BHLH transcription factor (MYC)-solute carrier family 7 member 5 (SLC7A5) axis in T cells, leading to impaired methionine uptake, reprogramming of one-carbon metabolism and epigenetic landscape, and ultimately promoting mitochondrial metabolic adaptivity and quiescence (Cheng et al., 2023; Oh et al., 2023). These potential targets further pave the way for possible therapeutic strategies targeting alkaliptosis to overcome resistance to immunotherapy.

## The function of alkaliptosis

**FIGURE 2:**
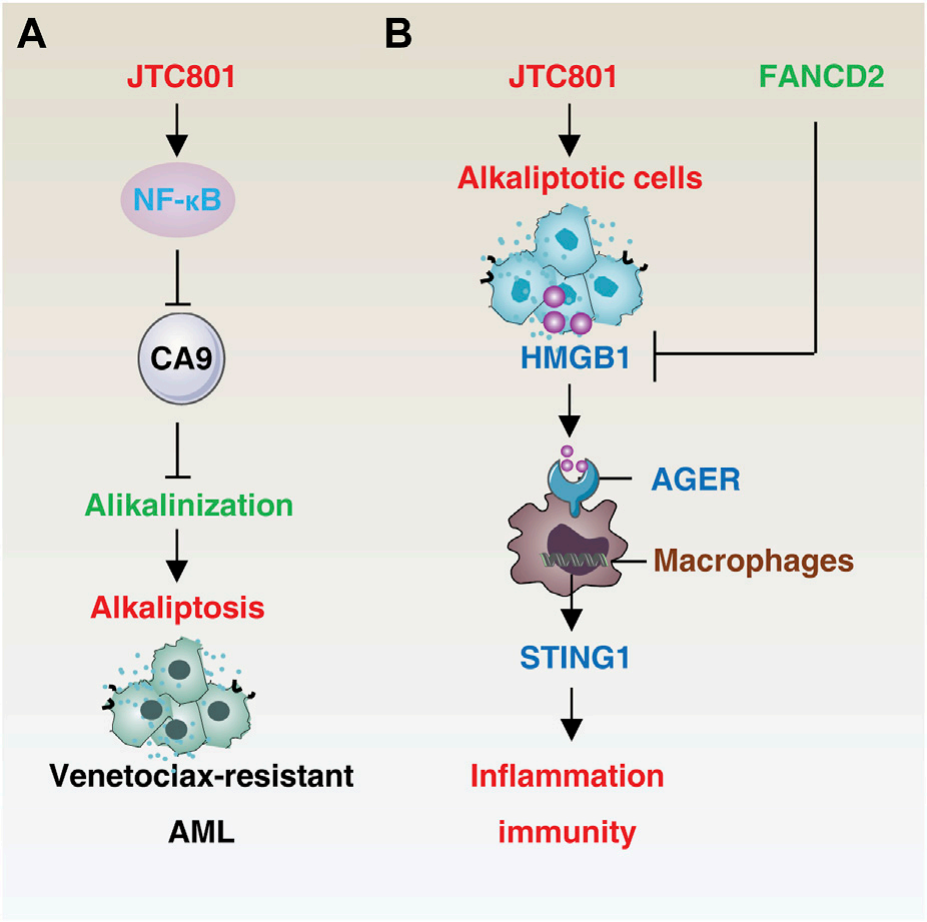
Mechanisms of alkaliptosis in drug resistance and immune response. **(A)** JTC801-induced alkaliptosis in venetoclax-resistant acute myeloid leukemia cells is mediated by the NF-κB-CA9 pathway. **(B)** Alkaliptotic cells secrete HMGB1, which binds to the receptor AGER of macrophages and triggers the STING1 pathway to initiate inflammation and an immune response. In contrast, FANCD2 suppresses HMGB1 release. Abbreviation. AML, acute myeloid leukemia; HMGB1, high mobility group box protein B1; AGER, advanced glycation end-product-specific receptor; STING1, stimulator of interferon response cGAMP interactor 1, FANCD2, FA complementation group D2.

The hallmarks of cancer constitute the organizing principles that enable the onset and progression of neoplastic disease (Hanahan, 2022). The induction of alkaliptosis can not only overcome the formation of multidrug resistance, but also plays an important role in tumor immunity and inflammatory response (Figure 2).

### Drug resistance

Drug resistance is a major contributor to treatment failure in cancer patients, resulting from multiple mechanisms (Szakacs et al., 2006). The plasma membrane V-ATPase-mediated reversal of pH blocks the accumulation of anticancer drugs in the tumor microenvironment and weakens the effectiveness of weak base or anthracycline drugs (Chen et al., 2022a). Anti-apoptotic BCL2 family members, such as BCL2 and BCL2-like 1 (BCL2L1), are also significant in the development of resistance. Inhibiting V-ATPase with bafilomycin A1 decreases expression of BCL2 and BCL2L1 and induces apoptosis in mouse Ms-1 cells in a caspase-independent manner (Sasazawa et al., 2009), though the clinical use of V-ATPase inhibitors remains controversial due to their unpredictable and non-tissue-specific side effects (Supino et al., 2008).

In the context of acute myeloid leukemia (AML), the induction of alkaliptosis has been shown to overcome BCL2-mediated resistance. Aberrant expression of BCL2 in AML is associated with decreased sensitivity to chemotherapy and increased recurrence rates (Campos et al., 1993; Döhner et al., 2017). Combination therapy with venetoclax (a BCL-2 selective inhibitor) and a hypomethylating agent or low-dose cytarabine has improved treatment outcomes in elderly or unfit AML patients (DiNardo et al., 2019; Wei et al., 2019; Maiti et al., 2021). However, relapse often occurs, leading to treatment failure due to the development of multidrug resistance. JTC801 has been shown to exhibit potent killing activity in venetoclax-resistant AML cells through activation of NF-κB-mediated CA9 downregulation (Zhu et al., 2021), offering a solution to overcome resistance caused by defective apoptosis. It is of interest to investigate if JTC801 can also overcome resistance to immune checkpoint inhibitors, which are a current frontline treatment approach for cancer patients.

### Immune consequences

The immunological specificity of the tumor microenvironment refers to the unique immune response that occurs within the microenvironment of a tumor (Khalaf et al., 2021). This includes the interactions between the cancer cells, immune cells, and the surrounding stroma that contribute to the development and progression of the tumor. The tumor microenvironment can suppress or alter the immune response, leading to immune evasion and tolerance of the cancer cells. Additionally, the presence of certain immune cell subtypes, such as regulatory T cells and myeloid-derived suppressor cells, can further contribute to the immunosuppressive state of the tumor microenvironment. Understanding the immunological specificity of the tumor microenvironment is crucial for developing effective immunotherapies for cancer treatment (Van Der Kraak et al., 2016; Kang et al., 2019).

The hypoxic and acidic microenvironment of solid tumors influences tumor immune surveillance (Chen G. et al., 2022). The presence of dead or dying cells in the tumor microenvironment can result in the release of damage-associated molecular patterns (DAMPs), which have both immunosuppressive and immunostimulatory effects on the immune response to the tumor (Krysko et al., 2012; Tang and Lotze, 2012; Tang et al., 2023b). For instance, ferroptotic PDAC cells can release KRAS-G12D protein, which drives tumor growth through polarization of macrophages (Dai et al., 2020), whereas the release of high mobility group box protein B1 (HMGB1) and membrane proteoglycan decorin (DCN) can stimulate anti-tumor cytotoxic T cell responses (Wen et al., 2019; Liu et al., 2022b). This dual function of DAMPs in both promoting and inhibiting tumor growth may be modulated by the same pattern recognition receptors (PRRs), with the strength of the signaling threshold determining the outcome.

HMGB1 is a widely studied DAMP that plays a role in various cell death-mediated inflammatory and immune responses (Chen R. et al., 2022; Chen et al., 2023; Tang et al., 2023c). In a resting state, HMGB1 is primarily located in the nucleus, where it acts as a DNA chaperone. JTC801-induced alkaliptosis of cancer cells leads to the release of HMGB1 into the cell supernatants (Fang et al., 2021). This release of HMGB1 from the nucleus involves nuclear DNA damage signaling and can be inhibited by the FA complementation group D2 (FANCD2)-dependent DNA repair pathways (Fang et al., 2021). Once released, extracellular HMGB1 binds to its receptor advanced glycosylation end-product specific receptor (AGER, also known as RAGE) in macrophages and activates the stimulator of interferon response cGAMP interactor 1 (STING1)-dependent production of pro-inflammatory cytokines, such as tumor necrosis factor (TNF) and interleukin 6 (IL6) (Fang et al., 2021). STING1 functions as a downstream effector molecule, amplifying the immune response to dying cells or pathogens, as well as mediating cell death and autophagy (Zhang et al., 2021; Zhang R. et al., 2022; Zhang RX. et al., 2022; Chen et al., 2022b). Dysregulation of STING1-related innate immune sensing has been implicated in various inflammatory diseases (Barber, 2015). Further research into the role of HMGB1, either alone or in combination with other DAMPs, in alkaliptotic cell death-mediated immune responses, including STING1 activation, could lead to the development of improved immunotherapeutic strategies.

## Conclusion and perspectives

Cancer cells undergo a metabolic shift characterized by an increased reliance on aerobic glycolysis, resulting in elevated production of lactate and protons (Cassim et al., 2020; Faubert et al., 2020; Pavlova et al., 2022). This acidification of the intracellular environment is countered by various acid removal mechanisms, resulting in a reversed pH compared to normal cells. This has prompted the investigation of pH modulation as a therapeutic strategy in cancer treatment (Ward et al., 2020). Recent studies have shown that intracellular acidification of cancer cells can induce multiple forms of cell death, including apoptosis and necroptosis (Furlong et al., 1997; Wang et al., 2015), whereas intracellular alkalization-dependent alkaliptosis is a novel form of cell death that has potential as a treatment for cancer, including drug-resistant cancers.

Despite recent progress in the cell death field, our knowledge of the underlying mechanisms of alkaliptosis remains limited. The positive regulators of alkaliptosis, such as NF-κB and STAT3, have been identified, however, the negative regulators and mediators of plasma membrane rupture remain to be characterized. Additionally, the metabolic changes accompanying cancer cells, including changes in fatty acid metabolism, have been linked to alkaliptosis, but the relationship between metabolic reprogramming and regulation of alkaliptosis is still not well understood. Further research is necessary to understand the regulation of pH at molecular, subcellular organelle, and tissue levels, especially the mechanisms by which pH regulation occurs *in vivo* and the role of the organelle network in alkaliptosis. It remains to be determined if physiological or pathological stress can induce alkaliptosis, and how pH changes can be dynamically monitored *in vivo* as markers of alkaliptosis. Furthermore, understanding the molecular connections and dynamic transformations between different forms of cell death is crucial for overcoming long-term drug resistance in tumors. Compared to other types of RCD, the research on alkaliptosis is still a young field and requires additional independent laboratories to confirm the key signals and mechanisms involved.
